# A Computational Model for Predicting RNase H Domain of Retrovirus

**DOI:** 10.1371/journal.pone.0161913

**Published:** 2016-08-30

**Authors:** Sijia Wu, Xinman Zhang, Jiuqiang Han

**Affiliations:** School of Electronic and Information Engineering, Xi’an Jiaotong University, Xi’an, China; "INSERM", FRANCE

## Abstract

RNase H (RNH) is a pivotal domain in retrovirus to cleave the DNA-RNA hybrid for continuing retroviral replication. The crucial role indicates that RNH is a promising drug target for therapeutic intervention. However, annotated RNHs in UniProtKB database have still been insufficient for a good understanding of their statistical characteristics so far. In this work, a computational RNH model was proposed to annotate new putative RNHs (np-RNHs) in the retroviruses. It basically predicts RNH domains through recognizing their start and end sites separately with SVM method. The classification accuracy rates are 100%, 99.01% and 97.52% respectively corresponding to jack-knife, 10-fold cross-validation and 5-fold cross-validation test. Subsequently, this model discovered 14,033 np-RNHs after scanning sequences without RNH annotations. All these predicted np-RNHs and annotated RNHs were employed to analyze the length, hydrophobicity and evolutionary relationship of RNH domains. They are all related to retroviral genera, which validates the classification of retroviruses to a certain degree. In the end, a software tool was designed for the application of our prediction model. The software together with datasets involved in this paper can be available for free download at https://sourceforge.net/projects/rhtool/files/?source=navbar.

## 1 Introduction

The retroviruses encompass a family of enveloped RNA-containing viruses that utilize reverse transcription of their genomes as an obligate step in virus replication [1]. Based on differences in morphological and biochemical features, retroviruses can be classified into seven genera including alpha-retrovirus, beta-retrovirus, gamma-retrovirus, delta-retrovirus, epsilon-retrovirus, lentivirus and spumavirus [2]. The common replication mode of all these retroviruses leads to reverse flow of genetic information from RNA template to intermediate DNA-RNA hybrid and then to complementary DNA [3]. The procedure thus contributes to virus propagation and influences genetic composition of infected cells [4]. To complete this process, reverse transcriptase enzyme is absolutely required which is encoded by polymerase (*pol*) gene [5]. As a key domain in reverse transcriptase, RNase H (RNH) specifically degrades the RNA strand of DNA-RNA replication intermediate to free the newly-made minus strand for use as a template in the synthesis of the plus strand of DNA [6–9].

The indispensable role of RNH in retroviral replication attracts the attention of some researchers. Their works reveal that inhibiting activity of RNH to break reverse transcription can be helpful to therapeutically intervene in some kinds of diseases, such as neoplasia, autoimmunity and immunosuppression [8–12]. Thus, RNH is qualified as an important and promising pharmaceutical target. But annotated RNHs in online database are not sufficient for statistical research. More new putative RNHs (np-RNHs) in the retroviruses are waiting for prediction with bioinformatics methods. However, the special RNH prediction software has been absent so far, and some classical database search tools [13–15] achieved unsatisfied accuracy rates which were lower than 80% in RNH recognition. Therefore, it is of great importance to come up with a computational RNH prediction model. This model will be conducive to reducing the number of amino acid sequences for biochemical experiment corroboration.

On the basis of amino acid sequences only, the proposed model recognizes start and end sites by SVM method to accomplish the aim of RNH prediction. The remainder of this paper is arranged as follows containing all the requirements of a sequence-based predictor [16, 17]. In Section 2, we will briefly describe the datasets used in this study. In Section 3, we will introduce the general scheme of our model and proper validation methods. Then the experimental results will be provided in Section 4, including the optimal parameters, validity analysis, RNH motif and evolutionary relationship. Finally, we will summarize our work and present the conclusions in Section 5.

## 2 Datasets

All the retroviral protein sequences involved in this work were collected from UniProt Knowledgebase (UniProtKB) [18, 19] and divided into two datasets. One named benchmark dataset contains 105 *pol* sequences with RNH annotations so as to complete the establishment of RNH prediction model. These data are all qualified to meet the criteria which stress RNH domains to be non-repetitive, manually annotated or reviewed and consistent in different databases. The other one includes 149,692 *pol* sequences and 320 non-*pol* sequences both without RNH annotations for predicting potential np-RNHs.

## 3 Methods

### 3.1 Prediction algorithm

#### Stage 1: Sample preparation

There are two sets of samples corresponding to start and end sites prediction respectively. The start samples are expressed as:

$$\begin{array}{l} ss=pol(i:i+L1-1)\;i=start(\mathrm{RNH})+d1\;d1=[-20,20]\\ =\left\{ \begin{array}{l} \mathrm{positive start sample if}\;d1=0\\ \mathrm{negative start sample if}\;d1\neq 0 \end{array}\right. \end{array},$$ (1)

while the end samples are given by:

$$\begin{array}{l} es=pol(i-L2+1:i)\;i=end(\mathrm{RNH})+d2\;d2=[-20,20]\\ =\left\{ \begin{array}{l} \mathrm{positive end sample if}\;d2=0\\ \mathrm{negative end sample if}\;d2\neq 0 \end{array}\right. \end{array}.$$ (2)

Here, *L*1 and *L*2 represent the length of start and end samples severally. *start*(RNH) and *end*(RNH) denote either true start and end site for a training *pol* sequence, or achieved start and end position after Smith-Waterman alignment [20] with training RNH domains for an inquired sequence.

#### Stage 2: Feature extraction

The widely recognized position weight matrix (PWM) [21–23] was employed to represent the conservative level of amino acid sequences in the benchmark dataset. By aligning positive start samples, negative start samples, positive end samples and negative end samples, PWMs are defined as:

$$\begin{array}{l} PWM_{\alpha ip}=\ln\frac{20^{k}\times (f_{\alpha ip}+\sqrt{N}/20^{k})}{\left(N+\sqrt{N}\right)}\;k=1,\;1\leq i\leq L\\ {}[p,L]=\left\{ \begin{array}{l} {}[1,L1]\;\text{for positive start samples}\\ {}[2,L1]\;\text{for negative start samples}\\ {}[3,L2]\;\text{for positive end samples}\\ {}[4,L2]\;\text{for negative end samples} \end{array}\right. \end{array}.$$ (3)

Here, *f*_*αip*_ refers to the absolute frequency of amino acid *α* in the *i*-th position of *N* aligned sequences for the *p*-th matrix. With these four matrixes, the scoring functions (*SF*) are given as follows:

$$SF_{p}=\sum_{i=1}^{L}PWM_{\alpha ip}.$$ (4)

The genuine start or end sample tends to have a larger value of *SF_p_* (*p* = 1 or 3) and a smaller value of *SF_p_* (*p* = 2 or 4).

#### Stage 3: SVM classifier

Support vector machine (SVM) [24] is a supervised algorithm based on statistical learning theory. Its basic idea is to construct an optimal hyperplane as the discriminative surface with the largest distance to the nearest training data points of any class. Like in some other publications [25–27], software LIBSVM [28] was used to fulfill the classification between positive start (end) samples and negative start (end) samples. LIBSVM can be obtained from the website http://www.csie.ntu.edu.tw/~cjlin/libsvm/.

#### Stage 4: RNH prediction

If the inquired sequence did not contain both of start and end site identified by SVM, it was inferred that no RNH domain exists in this sequence. Otherwise, the np-RNH could be predicted by the recognition of start and end site.

### 3.2 Performance assessment

The proposed RNH model was tested by jack-knife and n-fold cross-validation (10-fold CV or 5-fold CV) methods for an objective and comprehensive assessment. The quantitative evaluation results were measured by sensitivity (*Se*), specificity (*Sp*), overall accuracy (*Acc*) and Matthew’s correlation coefficient (*Mcc*). They are defined as follows:

$$\left\{ \begin{array}{l} Se=\frac{Tp}{Tp+Fn}\\ Sp=\frac{Tn}{Tn+Fp}\\ Acc=\frac{Tp+Tn}{Tp+Tn+Fp+Fn}\\ Mcc=\frac{Tp\times Tn-Fp\times Fn}{\sqrt{\left(Tp+Fn)(Tp+Fp)(Tn+Fn)(Tn+Fp\right)}} \end{array}\right.,$$ (5)

where *Tp* and *Fn* are the numbers of positive samples that are predicted to be positive and negative respectively, analogously, *Tn* and *Fp* are the numbers of negative samples that are predicted to be negative and positive respectively.

## 4 Results and Discussion

### 4.1 Parameter analysis

Two sets of parameters will be discussed in detail in this section. They are the range of candidate sites (*d*1, *d*2) and length of samples (*L*1, *L*2). The appropriate values of these parameters were trained by jack-knife and n-fold CV methods based on the benchmark dataset.

The absolute distances between positive sites and corresponding predicted sites by SW-alignment were counted for *d*1 and *d*2 parameters. They are all smaller than 20 amino acids (AA). Thus, samples prepared as formula (1) and formula (2) definitely contain all positive ones and a certain number of negative ones for a better classification by SVM method.

**Fig 1:**
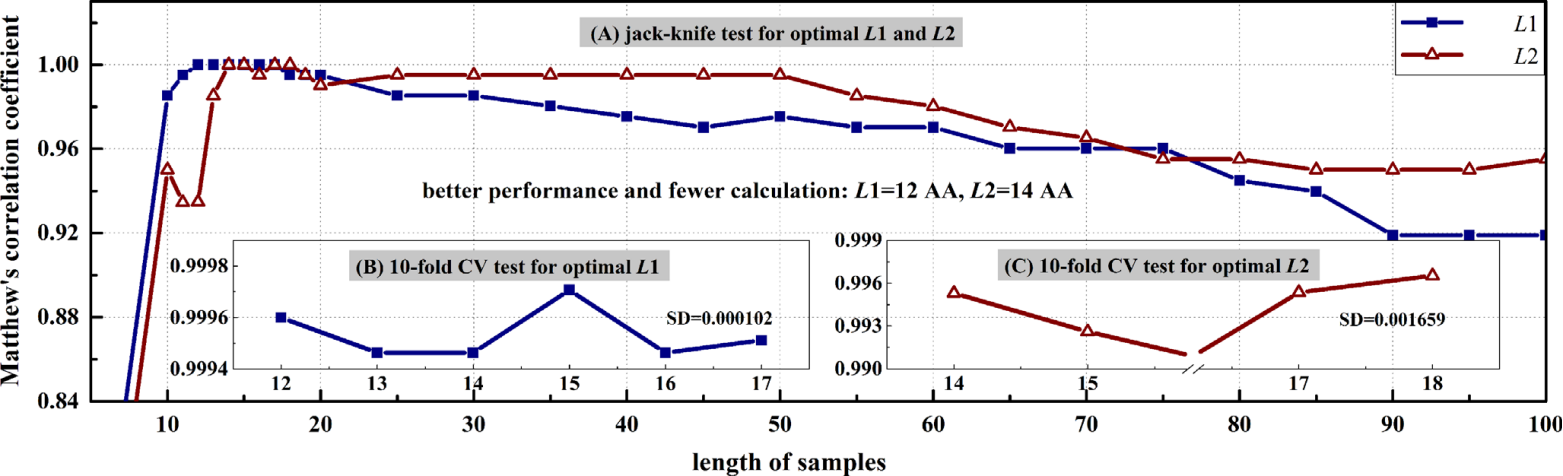
Change of average *Mcc* values versus different sample lengths. (A) Performance changes along with sample lengths from 10 AA to 100 AA under jack-knife test. (B)-(C) Performance varies along with optimal sample lengths selected in Fig 1(A) under 10-fold CV test. SD denotes the standard deviation of *Mcc* values.

The curves of *Mcc* versus different sample lengths were plotted in Fig 1 for *L*1 and *L*2 parameters. In this figure, *Mcc* values with *L*1 = 12 AA and *L*2 = 14 AA reach the peaks with fewer calculations under jack-knife test, as well as show very small differences from others under 10-fold CV test. Thus, the optimal *L*1 and *L*2 were set as aforesaid when taken into account both of the performance and computational amounts.

### 4.2 Validity analysis

**Fig 2:**
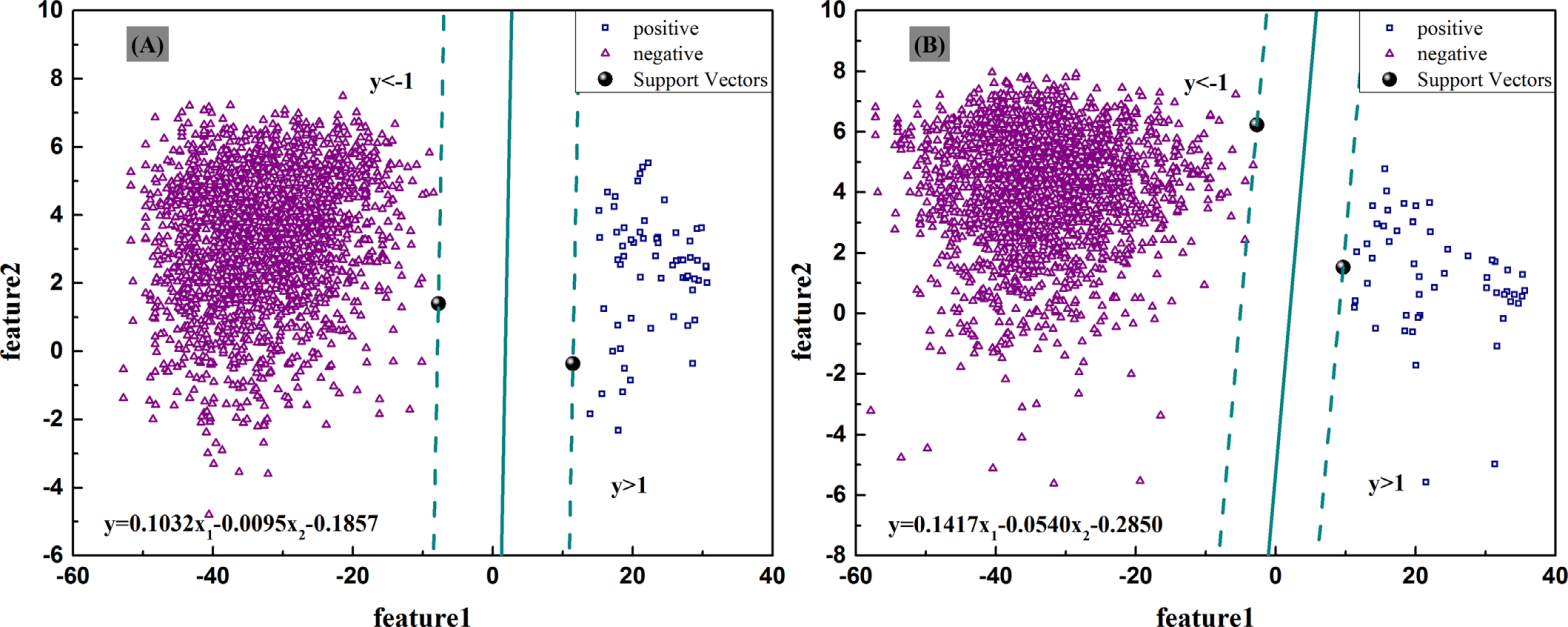
Classification results between positive and negative samples by SVM method. The solid lines represent the optimal classification hyperplanes constructed for (A) start samples and (B) end samples.

The proposed model has been tested from different aspects to demonstrate its validity in np-RNH prediction (Table 1, Fig 2). The first one is a test by increasingly used and widely recognized validation methods [16]. The classification accuracy rates are 100%, 99.01% and 97.52% respectively corresponding to jack-knife, 10-fold CV and 5-fold CV test. Another one is a comparison with three other classical database search tools. The differences are 20.95, 24.76, and 80.95 points after prediction accuracy of RNH model minus that of PSI-BLAST, CS-BLAST, and HMMER3 severally. The last one is a contrast of np-RNHs predicted in *pol* sequences and non-*pol* sequences. RNH model discovered 14,033 and zero np-RNHs after separately scanning 149,692 *pol* sequences and 320 non-*pol* sequences, which is in consistency with the existence of RNH as a domain in reverse transcriptase of *pol* [4, 8]. These results all confirm the validity of RNH model to a certain degree.

**Table 1:** Comparison results between RNH tool and three other classical database search tools.

Method	Train/test		Scan		
			Protein ^d^	number	np-RNHs
**RNH tool**	**5-fold CV**	*Acc* = 97.52% ^a^	*pol*	149,692	14,033
		*Se1* = 99.62%, *Sp1* = 100%, *Acc1* = 99.99%, *Mcc1* = 99.80% ^b^			
		*Se2* = 97.90%, *Sp2* = 100%, *Acc2* = 99.95%, *Mcc2* = 98.92% ^c^			
	**10-fold CV**	*Acc* = 99.01%			
		*Se1* = 99.91%, *Sp1* = 100%, *Acc1* = 99.998%, *Mcc1* = 99.96%	non-*pol*	320	0
		*Se2* = 99.08%, *Sp2* = 100%, *Acc2* = 99.98%, *Mcc2* = 99.53%			
	**jack-knife**	*Acc* = 100%			
	**self-consistency**	*Acc* = 105/105 = 100%			
**PSI-BLAST**	*Acc* = 83/105 = 79.05% (100%-79.05% = 20.95%)		-	-	-
**CS-BLAST**	*Acc* = 79/105 = 75.24% (100%-75.24% = 24.76%)		-	-	-
**HMMER3**	*Acc* = 20/105 = 19.05% (100%-19.05% = 80.95%)		-	-	-

^a^: the performance of RNH domain prediction.

^b^: the performance of start site prediction.

^c^: the performance of end site prediction.

^d^: the proteins without RNH annotations for scanning.

### 4.3 Sequence analysis

**Fig 3:**
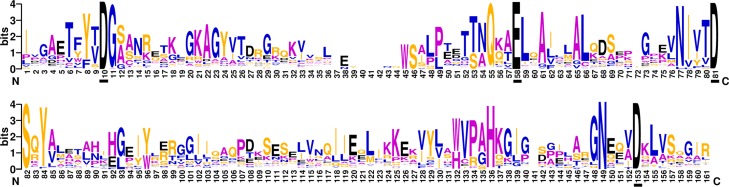
RNH motif plotted by weblogo software. The overall height of the stack indicates the sequence conservation at each position, the height of symbols within the stack represents the relative frequency of amino acid at that position, while the four underlined residues (D10, E58, D81 and D153) display the DEDD motif in RNH domain.

Weblogo software [29, 30] was employed to generate RNH motif in Fig 3. In this figure, the underlined D10, E58, D81 and D153 are four active-site residues based on 71 annotations of the benchmark dataset. These residues can be grouped into the DEDD motif, which coordinates with divalent metal ions to facilitate RNA hydrolysis during the catalytic process [31]. It is noteworthy that regions around DEDD motif are more conserved than others. The result supports the literatures [32, 33] that DEDD motif is essential for metal binding and catalytic activity. These conservativeness properties around D10 and D153 make it convenient to recognize start and end sites of RNH domains.

**Fig 4:**
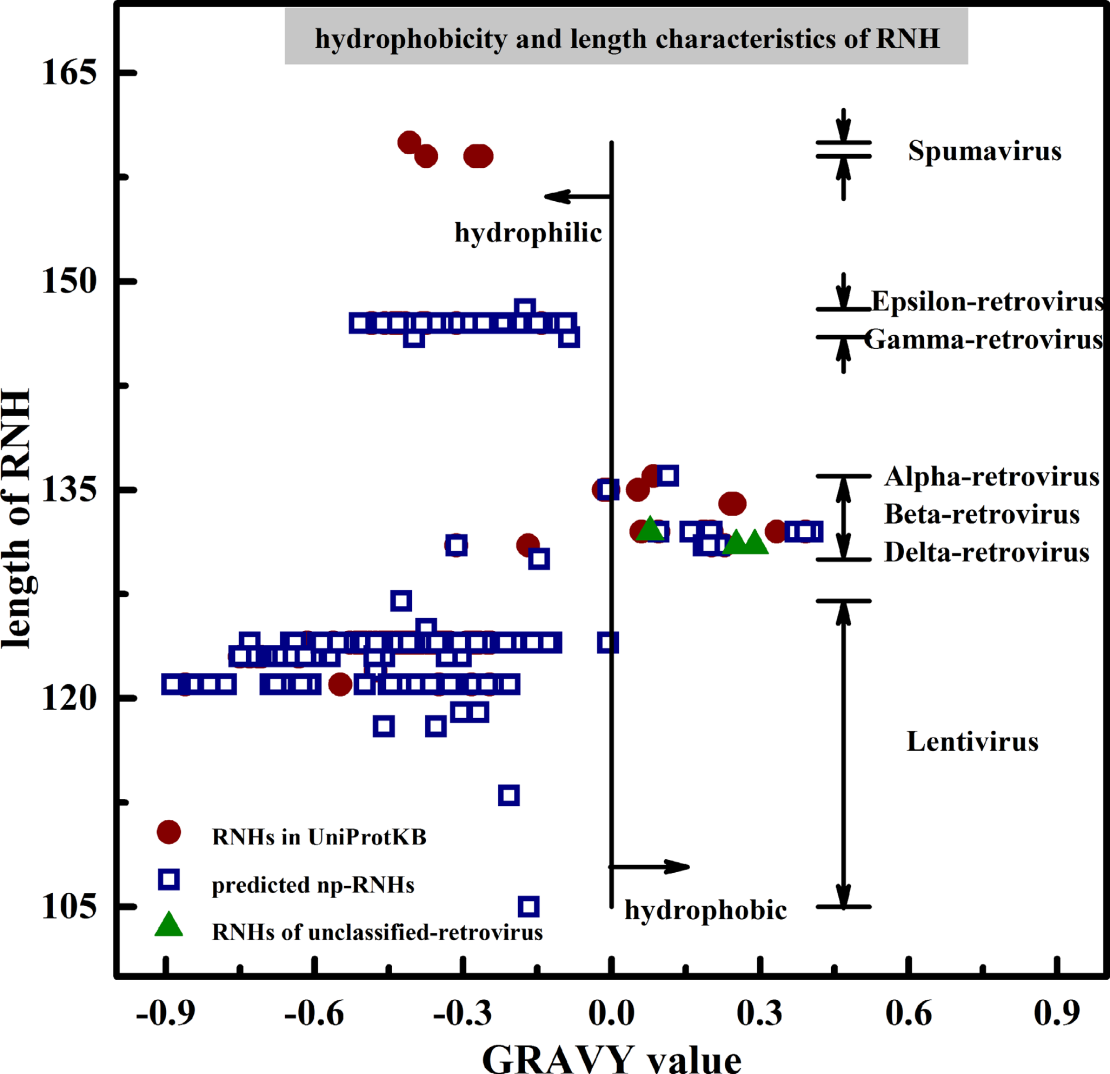
Hydrophobicity and length characteristics analyzed on RNH domains. RNHs in lentivirus are hydrophilic whose lengths range from 105 AA to 127 AA, RNHs in alpha-retrovirus, beta-retrovirus and delta-retrovirus are hydrophilic or hydrophobic whose lengths range from 130 AA to 136 AA, RNHs in epsilon-retrovirus and gamma-retrovirus are hydrophilic whose lengths range from 146 AA to 148 AA, while RNHs in spumavirus are hydrophilic whose lengths range from 159 AA to 160 AA.

Two properties were analyzed on RNH domains in Fig 4. One is length property and the other is hydrophobicity calculated by grand average of hydropathy (GRAVY) [34, 35]. This figure shows that RNHs in the same genus share particular length and GRAVY values. In other words, it can be concluded that length and hydrophobicity of RNH domains may have some relationships with retroviral genera. Based on the conclusion, Intracisternal A-particle (unclassified-retrovirus) may be a part of alpha-retrovirus, beta-retrovirus or delta-retrovirus. On the other hand, these two characteristics of RNH domains can be regarded as a corroboration of retroviral genera defined by *pol* and other elements [1, 2].

### 4.4 Evolutionary relationship analysis

**Fig 5:**
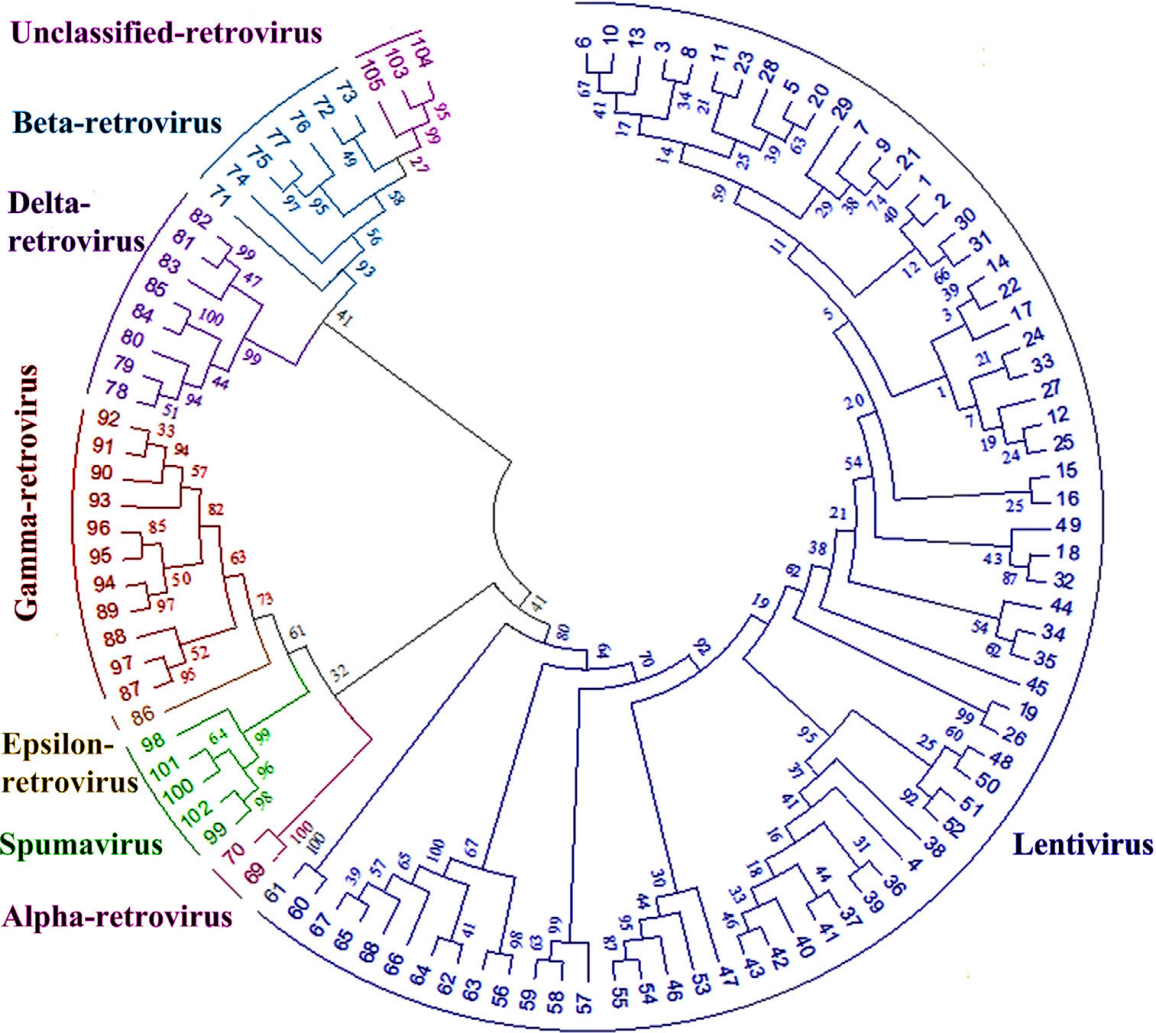
The evolutionary relationship in RNH of retroviruses. The leaf nodes denote annotated RNHs in the benchmark dataset, and the edge lengths describe the phylogenic relationship between these nodes.

MEGA software [36] was used to create the phylogenic tree by maximum likelihood method in Fig 5. This figure gives a comparison result between homology of RNHs within genera and that of inter-genera. It is expected and clear that RNHs in the same genus show higher homology than different genera. Thus, Intracisternal A-particle (unclassified-retrovirus) should be classified as beta-retrovirus, coinciding with analysis result in section 4.3. From another perspective, the evolutionary relationship in RNH of retroviruses can be seen as a technical validation of their classification in the literature [1], which uses *pol* sequence as one of the elements to define retroviral genera.

### 4.5 RNH prediction software

A software tool has been developed to further facilitate the application of proposed prediction model. It was implemented with C programing language for windows environment. This tool receives and sends files both with the format of FASTA. The input files contain inquired amino acid sequences, while the output files include the predicted np-RNHs. Each np-RNH annotation in export files indicates clearly its start and end site in the inquired sequence.

## 5 Conclusion

A computational RNH model was put forward in this paper based on amino acid sequences only with high classification accuracy. This model discovered 14,033 np-RNHs after scanning sequences without RNH annotations. Based on these predicted np-RNHs and annotated RNHs, a preliminary experiment was performed to analyze the length, hydrophobicity and evolutionary relationship of RNH domains. The results indicate a correlation between these three characteristics and retroviral genera, which confirms the classification of retroviruses to some extent. To further facilitate the application of our proposed model, software named RNHtool has been developed.
