# Correction: A Computational Model for Predicting RNase H Domain of Retrovirus

**DOI:** 10.1371/journal.pone.0165216

**Published:** 2016-10-18

**Authors:** Sijia Wu, Xinman Zhang, Jiuqiang Han

The financial disclosure and author contributions for funding acquisition are incomplete and cannot reflect the fact of financial support for this publication.

The correct financial disclosure is: This work was supported by grants from the Ph.D. Program Foundation of the Ministry of Education of China (No. 20110201110010), the National Natural Science Foundation (No. 61673316), the Major Science and Technology Foundation in Guangdong Province of China (No. 2015B010104002), the Fundamental Research Funds for the Central Universities, and the Natural Science Foundation of Shaanxi Province (No. 2015JM6336). The funders had no role in study design, data collection and analysis, decision to publish, or preparation of the manuscript.
